# A One Health investigation of a Crimean-Congo Hemorrhagic fever outbreak reveals high seropositivity in livestock in Lyantonde District, Uganda, 2024

**DOI:** 10.1016/j.onehlt.2026.101470

**Published:** 2026-06-10

**Authors:** Luke Nyakarahuka, Sophia Mulei, Shannon L. Whitmer, Joanita Mutesi, Jimmy Baluku, Calvin Richie Torach, Diana Namanya, Alex Tumusiime, Jackson Kyondo, Carson T. Telford, Amy Schuh, Joel M. Montgomery, Julius J. Lutwama, Stephen K. Balinandi, Trevor R. Shoemaker

**Affiliations:** a Uganda Virus Research Institute (UVRI), Entebbe, Uganda; b Department of Biosecurity, Ecosystems and Veterinary Public Health, College of Veterinary Medicine, Animal Resources and Biosecurity (COVAB), Makerere University, Kampala, Uganda; c U.S. Centers for Disease Control and Prevention (CDC), Atlanta, Georgia, USA

**Keywords:** Crimean-Congo Hemorrhagic fever, Uganda, One health, Zoonotic disease, Seroprevalence, Molecular epidemiology

## Abstract

In January 2024, an outbreak of Crimean-Congo hemorrhagic fever (CCHF) was confirmed in Buyanja village, Lyantonde District, Uganda. Initially suspected to be anthrax, laboratory tests at the Uganda Virus Research Institute (UVRI) confirmed CCHF, prompting a One Health response. Five (5) human cases were confirmed through both Reverse Transcription Polymerase Chain Reaction(RT-PCR) and IgM Enzyme-Linked Immunosorbent Assay (ELISA) testing, all of which were associated with direct contact with livestock, particularly goats. Among the twenty-six goats sampled, 88% (23/26) tested positive for CCHFV-specific IgG antibodies, indicating prior exposure. All the goat samples were negative by PCR, suggesting no active infection at the time of testing. Molecular sequencing revealed viral strains clustering in the Africa II clade, with multiple S-segment lineages circulating. These findings support localized virus persistence in Lyantonde area and highlight occupational exposure risks in Uganda's cattle corridor. Strengthening One Health surveillance with emphasis on veterinary services integration is critical to CCHF control.

## 1 Introduction

Crimean-Congo hemorrhagic fever (CCHF) is a zoonotic tick-borne viral disease caused by a member of the *Orthonairovirus* genus, *Nairoviridae* family. The disease carries a high case fatality rate of up to 40%, and is characterized by fever, myalgia, and hemorrhagic symptoms [1]. Human exposure to the virus is often occupational, occurring through contact with infected animals, carcasses, or tick bites. Person-to-person transmission has also been reported, especially nosocomial infections [2].

Subsequent outbreaks of CCHF have been reported in Uganda, with most cases concentrated along the cattle corridor [1], [3], [4], [5]. These outbreaks have predominantly affected individuals involved in the livestock sector, including herdsmen, veterinarians, animal handlers, and abattoir workers. Seroprevalence studies have demonstrated notable antibody prevalence among domestic animals, with prevalence of 16.9% in cattle, 49.2% in sheep, and 48.7% in goats [6]. Small ruminants and goats, in particular, have consistently shown the highest seropositivity levels, as documented in multiple Ugandan studies [4], [6]. The primary tick vector implicated in CCHF transmission in Uganda is *Rhipicephalus* spp. [7], [8], [9]; however, CCHFV RNA has also been detected in other tick genera such as *Amblyomma*, indicating their potential role in virus transmission [3], [9]. The epidemiology of CCHF in Uganda is still evolving. Although risk maps informed from serological livestock studies have predicted the highest levels of seroprevalence to be in northern regions of the country [10], sporadic outbreaks of incident human cases continue to occur in central and southwestern districts in the cattle corridor. In January 2024, we investigated suspected anthrax-related deaths in Lyantonde district that were later confirmed as CCHF. Our findings highlight this persistent and geographically unpredictable nature of CCHF in Uganda and discordance between the geographic distribution of elevated livestock seroprevalence compared to human incidence.

## 2 Materials and methods

### 2.1 Study design, case definition and sampling strategy

This was a descriptive One Health outbreak investigation conducted following laboratory confirmation of Crimean-Congo hemorrhagic fever virus (CCHFV) infection in Lyantonde District, Uganda. The investigation integrated human case investigation, targeted livestock sampling, laboratory testing, molecular characterization, and community engagement. It was implemented as part of the national public health emergency response under the approval and coordination of the National Task Force.

The investigation was conducted from February 19 to 29, 2024, in Lyantonde District, Uganda. A suspected human case of Crimean-Congo hemorrhagic fever (CCHF) was defined as any person presenting with fever and signs of acute illness such as diarrhea, vomiting, abdominal pain, headache, muscle and joint pain, hiccups with or without bleeding. Initial cases were first suspected to be anthrax because patients presented with profuse bleeding and poor clot formation, including persistent bleeding at injection sites. Samples were therefore initially referred to the Anthrax Diagnostic Laboratory at the Uganda Virus Research Institute (UVRI) in Arua, where anthrax testing returned negative results. Given the continued bleeding manifestations, further investigations were undertaken to evaluate other potential causes of hemorrhage, including viral etiologies. Based on the clinical presentation particularly unexplained bleeding, a hallmark feature of viral hemorrhagic fevers (VHFs) the cases were subsequently investigated as suspected VHF infections. Confirmed cases were those that tested CCHFV positive by RT-PCR and/or IgM ELISA. After laboratory confirmation of index cases, additional human samples were collected from symptomatic individuals and close contacts primarily family members and neighbors in Kasagama subcounty, buyanja Village, Lyantonde District.

Because this was an outbreak investigation, sampling was purposive and targeted rather than random, convenience-based, or cluster-based. Human sampling focused on suspected cases, symptomatic individuals, and close contacts identified through field investigation in the affected village. Animal sampling was conducted in households linked to confirmed human CCHF cases, with the objective of investigating possible sources of infection and assessing evidence of CCHFV exposure among livestock kept in affected homes. Livestock were eligible for sampling if they were owned by households with confirmed human cases and were present at the time of the investigation. In the affected households visited, goats were the only livestock species available for sampling; therefore, animal sampling was restricted to goats. No formal sample size calculation was conducted because the investigation was implemented as part of an emergency public health outbreak response. The number of animals sampled was determined by the livestock available in affected households during the field investigation. In total, 8 human samples were collected. Simultaneously, animal samples were collected from 26 goats residing in households of confirmed human cases who owned only goats. Livestock sampling was conducted approximately one month after confirmation of the first human CCHF case. The first human case was confirmed on 19 January 2024, and goat sampling was conducted on 19 February 2024 in households linked to confirmed human cases. Therefore, animal sampling was conducted after human case confirmation and was intended to assess livestock exposure and local CCHFV circulation in affected households.

### 2.2 Community engagement

**Fig. 1:**
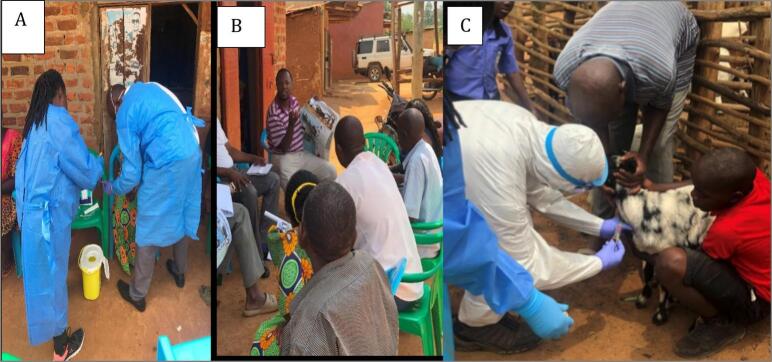
**Field activities during the CCHF outbreak investigation.** Plate A shows human sample collection conducted in the affected home by UVRI laboratory staff. Plate B shows community education using a CCHF fever poster detailing transmission and preventive measures against CCHFV. Plate C shows animal sample collection in a goat from one of the homes of a confirmed case.

Field activities included both human and animal sampling, as well as community engagement. As shown in Fig. 1, healthcare workers in appropriate personal protective equipment (PPE) collected blood samples from symptomatic individuals and high-risk contacts at their homes. Concurrently, veterinary staff sampled goats from affected households. Community sensitization was conducted through village meetings, where public health officials delivered health education and distributed printed materials to raise awareness about CCHF transmission and prevention.

### 2.3 Laboratory testing

The serological assays used in this investigation were U.S. CDC in-house ELISA platforms that have been used in previous CCHF outbreak investigations and seroepidemiologic studies in Uganda and comparable settings [1], [3], [4], [5], [6], [11]. Human sera were tested using an IgM-capture ELISA to identify recent CCHFV infection, whereas goat sera were tested using an IgG ELISA to assess prior CCHFV exposure. Goat IgM testing was not performed. Therefore, goat IgG seropositivity was interpreted as evidence of previous exposure and local virus circulation, but not as evidence of active infection or current viremia. Active infection was assessed using RT-PCR targeting the CCHFV S-segment, and all goat samples were negative by RT-PCR. ELISA testing included positive and negative control sera and antigen controls in each run. RT-PCR assays included extraction controls, positive controls, negative controls, and no-template controls. RT-PCR-positive human samples were further characterized by sequencing.

Laboratory confirmation of suspected CCHF cases was conducted at the Uganda Virus Research Institute (UVRI). RNA was extracted from samples using MagMAX RNA Isolation Kits (Thermo Fisher Scientific, Vilnius, Lithuania) following the manufacturer's protocol. RT-PCR targeting the CCHFV S-segment was performed using the Luna® Universal Probe One-Step RT-qPCR Kit (NEW ENGLAND Biolabs, Beverly, MA) with in-house primers and a probe (Forward: CCHFV-S-4-F: CAA AGA AAC ACG TGC CGC TT, Reverse: CCHFV-S-79-R: ATT CAC CTC GAT TTT GTT TTC CAT, Probe: CCHFV-S-24-Prb: 5′ 6-FAM-AC GCC CAC A/ZEN quencher/G TGT TCT CTT GAG TGT TAG CA-3′). These primers were adapted from previously published protocols [1], [11]. RT-PCR positive samples were subjected to sequencing following established procedures as published previously [1], [5], [11].

CCHF-specific human IgM antibodies were measured using a U.S. CDC in-house IgM-capture ELISA, following established protocols [12]. Serum samples were captured on plates coated with anti-human IgM antibodies and reacted with CCHFV and control antigens. Detection was performed using horseradish peroxidase (HRP)–conjugated anti-mouse IgM and ABTS substrate. Optical density was read at 490 nm using a BioTek PowerWave 340 plate reader, and samples with a summed optical density (OD_Sum_) ≥ 0.45 were considered positive.

Goat sera were tested for anti-CCHFV IgG using an in-house ELISA developed by the CDC, as previously described [4] Briefly, microtiter plates were coated with CCHFV-specific capture antibody, incubated with CCHFV antigen and control antigen, and then with serially diluted test sera and positive and negative controls. Detection was performed using HRP-conjugated anti-goat IgG and ABTS substrate. Results were read at 490 nm, and samples with a sum OD (ODSum) ≥ 0.95 were considered positive.

Whole-genome sequencing was conducted on RT-PCR positive samples using next-generation sequencing (NGS) platforms at UVRI, in collaboration with the CDC Viral Special Pathogens Branch.

### 2.4 Data analysis

Data were entered and cleaned in Microsoft Excel and analyzed using RStudio version 4.3.3. Because this was a descriptive outbreak investigation with a small, purposively selected sample, analyses were limited to descriptive summaries. Frequencies, proportions, and exact 95% confidence intervals were calculated for key findings, including the proportion of goats seropositive for CCHFV-specific IgG antibodies. No statistical hypothesis testing was performed because the investigation was not designed to compare groups or test associations. Adjustment for herd-level clustering was not performed because livestock findings were presented descriptively and no inferential comparisons were made. Multivariable analysis was also not conducted because of the small number of sampled animals and the limited number of seronegative goats, which would have produced unstable and unreliable model estimates. Maps were generated using QGIS software version 3.30.3.

### 2.5 Ethical considerations

Verbal informed consent was obtained from all participants prior to sample collection. The investigation was conducted under the mandate of the national epidemic response, in accordance with the Uganda Ministry of Health's emergency public health response protocols. As such, institutional review board (IRB) approval was not required. Participant confidentiality and privacy were strictly maintained throughout the investigation.

## 3 Results

### 3.1 Human cases

**Fig. 2:**
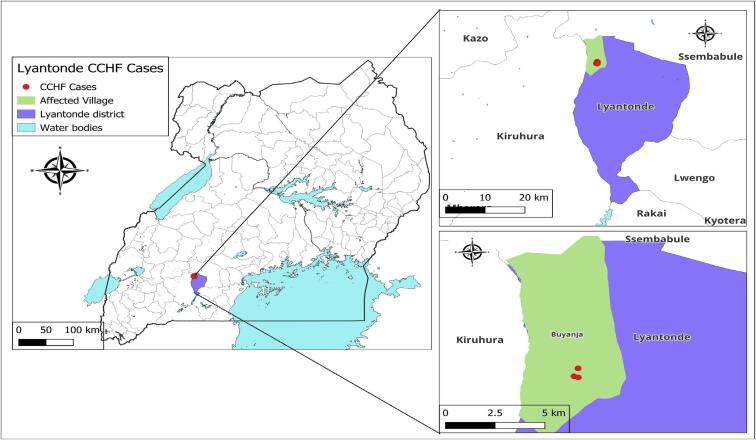
The location of Lyantonde District and the affected village, situated in the mid-southwestern cattle corridor of Uganda.

Fig. 2 displays the spatial distribution of confirmed CCHF cases in Lyantonde District, southwestern Uganda. The map highlights the affected village of Buyanja, located near the border with Ssembabule, Kazo, and Kiruhura districts, all of which have reported incident human CCHF cases previously. All confirmed cases originated from this single village, clustered within a narrow geographical area (∼5 km radius), suggesting localized transmission. The district lies within Uganda's cattle corridor which is a high-risk zone for tick-borne zoonoses due to intensive livestock farming. From our investigation, a total of five recent human CCHF cases were confirmed in Lyantonde District. Three cases tested positive by RT-PCR and two by IgM ELISA. In addition, two individuals tested positive for CCHFV IgG, indicating past exposure. All confirmed cases were males aged 34 to 47 years, primarily engaged in butchering and livestock handling. Symptoms included fever, vomiting, generalized weakness, and hemorrhagic manifestations. One additional probable case presented with compatible symptoms and exposure history but tested negative for CCHFV RNA, IgG and IgM antibodies. The likely source of infection was close contact with infected animals or carcasses; however, person-to-person transmission could not be ruled out.

### 3.2 Animal findings

None of the 26 goats sampled from case households tested positive for CCHFV by RT-PCR. However, 88.5% of goats were seropositive for anti-CCHFV IgG antibodies (23/26; 95% CI: 69.8–97.6%), indicating prior exposure to CCHFV. Most sampled goats were local breed, middle-aged, and showed no clinical signs of illness at the time of sampling. Ticks were observed on 96.2% of goats (25/26; 95% CI: 80.4–99.9%).

### 3.3 Molecular epidemiology

**Fig. 3:**
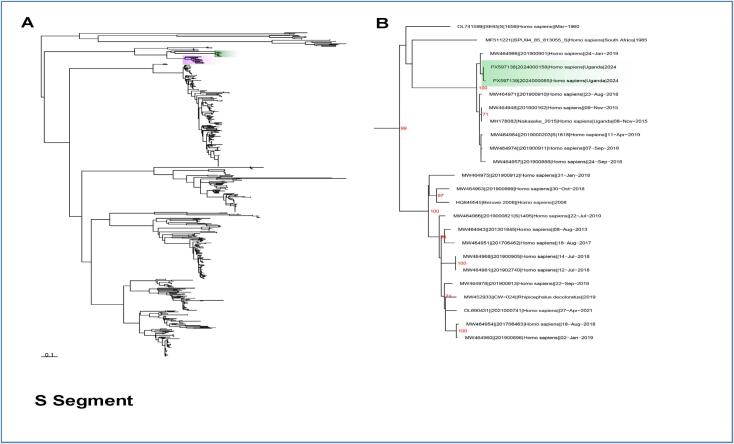
**Phylogenetic analysis of CCHFV S-segment sequences from the 2024 Lyantonde outbreak.** A) Inferred evolutionary relatedness (maximum likelihood) using all available full-length CCHFV S-segments. Recent CCHFV sequences from Uganda (2016+) form two clades (highlighted purple or green).B) Expanded Uganda-specific clade (highlighted green in Panel A) demonstrates the relationship between the Lyantonde sequences (highlighted light green) and recent sequences from Uganda, including the two Lyantonde outbreak sequences deposited under accession numbers PX597138 (sample 2024000158_S) and PX597139 (sample 2024000085_S)**.** (For interpretation of the references to colour in this figure legend, the reader is referred to the web version of this article.)

Fig. 3 presents the phylogenetic analysis of CCHFV sequences from confirmed human cases. Viral sequences clustered within the Africa II clade, with S-segment analysis revealing the circulation of at least two distinct viral lineages. Comparison with historical sequences [1], [5], [11] indicates low genetic divergence, suggesting long-term persistence and limited evolution of CCHFV strains in the Ugandan ecosystem. Two sequences from the Lyantonde outbreak;PX597138 (2024000158_S) and PX597139 (2024000085_S)represent the S-segment genomes from the two individual samples and were most closely related to the 2019 Kiruhura sequence (MW464966) (Fig. 2). The corresponding M- and L-segment sequences for the same two samples, included in the supplementary materials (PX597140 / 2024000158_M and PX597141 / 2024000085_M; PX597142 / 2024000158_L and PX597143 / 2024000085_L), also clustered with MW464966, with bootstrap support values of 77% and 100% for the M and L segments, respectively.

## 4 Discussion

This investigation confirms ongoing transmission of Crimean-Congo hemorrhagic fever virus (CCHFV) in Uganda's cattle corridor, with Lyantonde District emerging as a significant hotspot. Consistent with earlier reports from Kiruhura, Nakaseke, and Kyotera districts, the present outbreak reaffirms the endemicity of CCHFV in southwestern Uganda, driven by frequent human-livestock-tick interactions [4], [5], [6], [7], [8], [11]. The ecological positioning of Lyantonde along key livestock trade routes connecting Uganda to regional markets and bordering countries of Rwanda, Tanzania, and Democratic Republic of Congo may further facilitate cross-border movement of infected livestock and vectors, amplifying transboundary risk. The consistent occupational link between confirmed human cases and livestock butchering or handling underscores the heightened vulnerability among men in animal-related trades. This pattern mirrors previous outbreaks in Uganda and globally [13], where occupational exposure remains the primary route of human infection.

Of note, the high IgG seroprevalence (88%) observed in goats, despite the absence of active viremia, echoes findings from previous studies, which also reported high CCHFV seroprevalence in goats [7], [14] and highlighted their role as reliable sentinels of past viral circulation. Several structural and behavioral factors may explain this trend. Compared to cattle, goats often receive less attention in tick control programs, are less likely to be dipped or sprayed, and frequently share human dwellings at night for protection against theft, thereby increasing human-vector proximity and spillover risk. This structural oversight in vector control strategies targeting small ruminants could be silently sustaining CCHFV enzootic cycles in peri-domestic settings.

Furthermore, previous research suggests that CCHFV infections in goats may adversely affect reproductive health, including increased rates of abortion and stillbirths [6] compounding the economic burden on smallholder farmers. These findings call for tailored, affordable tick-control strategies for goats such as farmer-friendly spray races or portable pressure pumps to reduce tick loads and subsequent human risk.

The high CCHFV IgG seroprevalence observed among goats should be interpreted as evidence of prior exposure and intense local virus circulation within the livestock-tick-human interface, rather than direct evidence of active infection in the sampled animals. This interpretation is supported by the absence of detectable CCHFV RNA in all goat samples by RT-PCR. Therefore, while the goat findings do not prove that the sampled animals were the immediate source of human infection, they provide important evidence that livestock in affected households had been exposed to CCHFV and that the ecological conditions required for virus maintenance were present in the area.

The public health relevance of this finding lies in the close contact between humans, livestock, and ticks in affected households. All confirmed human cases had histories of livestock handling or butchering, suggesting that occupational and peri-domestic exposure may have contributed to spillover risk. High livestock seropositivity in this setting therefore indicates a potentially elevated risk environment for humans, particularly among butchers, animal handlers, herders, and household members involved in animal care.

Several ecological and husbandry-related factors may have contributed to CCHFV circulation in this outbreak setting. Lyantonde District lies within Uganda's cattle corridor, where frequent livestock movement, animal trade, and close human-livestock contact may facilitate maintenance and spread of tick-borne pathogens. The observation of ticks on most sampled goats further supports the plausibility of vector-mediated CCHFV transmission in affected households. In addition, livestock movement through local and regional trade networks, including movement across district and national borders, may contribute to the introduction or persistence of infected ticks and exposed animals. However, because ticks were observed but not systematically collected or tested during this investigation, the specific tick species involved and their infection status could not be confirmed.

Molecular sequence analysis strengthens the known geographic distribution of CCHFV clades in Uganda. All sequenced isolates clustered within the Africa II clade, confirming the localized and persistent nature of CCHFV strains in Uganda. These results are consistent with low genetic diversity and slow molecular evolution reported for Ugandan CCHFV strains [1], [8]. The consistent clustering of Lyantonde isolates with previously reported Africa II sequences from central and southwestern Uganda reinforces the notion of endemic virus reservoirs. Limited sequence divergence across the S, M, and L segments suggests constrained molecular evolution possibly due to stable host-vector cycles. This stability, combined with sporadic human spillovers, mirrors trends observed in endemic foci across East Africa [1], [8].

Serologic findings from human cases, including detection of both IgM and IgG antibodies, point to active and past infections, respectively. The presence of survivors with IgG positivity further supports the hypothesis that CCHF incidence in Uganda is underreported. This aligns with prior observations that acute hospital-diagnosed cases represent only the “tip of the iceberg,” while a larger, unrecognized burden persists in the community due to limited diagnostics, misclassification as malaria or other febrile illnesses, and low public awareness [15], [16].

Community awareness of CCHF remains a critical gap. Despite sensitization efforts during this outbreak, there remains widespread misunderstanding and limited local vocabulary to describe CCHF, complicating risk communication. The absence of culturally adapted terms for CCHF makes the disease abstract and unfamiliar to at-risk populations. Without sustained health education and community engagement, outbreaks may continue to fuel fear, stigma, or attribution to supernatural causes.

This investigation applied a One Health approach by integrating human case investigation, targeted livestock sampling, laboratory diagnostics, molecular epidemiology, community engagement, and assessment of the livestock-tick-human interface. The human investigation confirmed recent CCHFV infection among persons with livestock-related exposures, while animal testing demonstrated high CCHFV IgG seropositivity among goats from affected households. Environmental and vector-related observations, particularly the presence of ticks on most sampled goats and the location of the outbreak within Uganda's cattle corridor, provided additional context for interpreting transmission risk. Together, these findings indicate that CCHF prevention and control in this setting requires coordinated action across public health, veterinary, community, and vector-control systems.

Our findings reinforce the importance of the One Health approach integrating human, animal, and environmental health systems in surveillance and outbreak response. As zoonotic spillovers escalate across Africa, investments in integrated tick control, regular animal screening, personal protective equipment (PPE) for high-risk occupations, and multisectoral coordination are imperative.

This investigation had some limitations. Livestock sampling was purposive and focused on households linked to confirmed human CCHF cases; therefore, the findings should be interpreted as evidence of exposure in affected households rather than as district-level livestock seroprevalence. Because animal sampling was conducted at one time point, approximately one month after confirmation of the first human case, the timing of livestock exposure could not be determined precisely. Goat IgG seropositivity was interpreted as evidence of prior CCHFV exposure, while RT-PCR negativity indicated no detectable active infection at the time of sampling. Although the U.S. CDC in-house CCHFV ELISA used in this investigation has been applied in previous studies, possible serological cross-reactivity cannot be completely excluded. Despite these limitations, the combined human, animal, laboratory, and epidemiological findings provide important evidence of CCHFV circulation in affected households and highlight the value of One Health outbreak investigations.

In conclusion, the 2024 CCHF outbreak in Lyantonde District highlights the persistence and complexity of CCHFV transmission in Uganda's cattle corridor. High goat seroprevalence, low viral diversity, and occupationally linked human infections emphasize the need for enhanced surveillance, tailored tick control for small ruminants, and community-centered education. A strengthened One Health framework connecting veterinary, public health, and environmental sectors is critical for mitigating future outbreaks and reducing the zoonotic burden of CCHFV.

## CRediT authorship contribution statement

**Luke Nyakarahuka:** Writing – review & editing, Writing – original draft, Visualization, Validation, Supervision, Software, Resources, Project administration, Methodology, Investigation, Formal analysis, Data curation, Conceptualization. **Sophia Mulei:** Writing – review & editing, Writing – original draft, Visualization, Validation, Software, Methodology, Investigation, Formal analysis, Data curation, Conceptualization. **Shannon L. Whitmer:** Writing – review & editing, Visualization, Validation, Supervision, Software, Resources, Methodology, Funding acquisition, Formal analysis, Data curation, Conceptualization. **Joanita Mutesi:** Writing – review & editing, Writing – original draft, Visualization, Methodology, Investigation, Formal analysis, Data curation, Conceptualization. **Jimmy Baluku:** Writing – review & editing, Visualization, Methodology, Investigation, Formal analysis, Data curation. **Calvin Richie Torach:** Writing – review & editing, Methodology, Investigation, Formal analysis, Data curation. **Diana Namanya:** Writing – review & editing, Methodology, Investigation, Data curation. **Alex Tumusiime:** Writing – review & editing, Validation, Supervision, Methodology, Investigation, Formal analysis, Data curation. **Jackson Kyondo:** Writing – review & editing, Visualization, Validation, Methodology, Investigation, Data curation. **Carson T. Telford:** Writing – review & editing, Visualization, Validation, Software, Methodology, Formal analysis, Data curation. **Amy Schuh:** Writing – review & editing, Validation, Supervision, Resources, Project administration, Methodology, Funding acquisition. **Joel M. Montgomery:** Writing – review & editing, Validation, Supervision, Resources, Project administration, Methodology, Funding acquisition, Conceptualization. **Julius J. Lutwama:** Writing – review & editing, Validation, Supervision, Resources, Project administration, Methodology, Funding acquisition, Conceptualization. **Stephen K. Balinandi:** Writing – review & editing, Validation, Supervision, Resources, Project administration, Methodology, Investigation, Funding acquisition, Data curation, Conceptualization. **Trevor R. Shoemaker:** Writing – review & editing, Visualization, Validation, Supervision, Resources, Project administration, Methodology, Investigation, Funding acquisition, Data curation, Conceptualization.

## Ethics approval

This investigation was conducted as part of the national public health emergency response and adhered to institutional and national ethical guidelines approved by the Uganda Ministry of Health National Task Force for Crimean-Congo Hemorrhagic Fever. Verbal informed consent was obtained for all human participants and for animal sampling.

## Declaration of competing interest

No conflicts of interest to declare. This manuscript has not been previously published and is not under consideration elsewhere. No financial interests are associated with the content. This manuscript was prepared with the assistance of OpenAI's ChatGPT (v4, March 2024), used to support editing, formatting, and summarization. All content was verified and approved by the authors.

The authors declare that they have no known competing financial interests or personal relationships that could have appeared to influence the work reported in this paper.

The findings and conclusions in this manuscript are those of the authors and do not necessarily represent the official position of the Uganda Virus Research Institute or the U.S. Centers for Disease Control and Prevention.

No specific funding was received for this investigation beyond routine support for national public health emergency response activities.

## Data Availability

Data will be made available on request.

## Appendix A Supplementary data

Maximum-likelihood Trees of the M and L segments showing Lyantonde CCHF Virus Sequences within Africa 11.
