# Supplementary material for: A One Health investigation of a Crimean-Congo Hemorrhagic fever outbreak reveals high seropositivity in livestock in Lyantonde District, Uganda, 2024
Source: One Health. 2026 Jun 10;23:101470. doi: 10.1016/j.onehlt.2026.101470 (PMC13279415; doi:10.1016/j.onehlt.2026.101470)

**Supplementary Figure 1.** Maximum-likelihood tree of the M-segment showing Lyantonde sequences within Africa II.


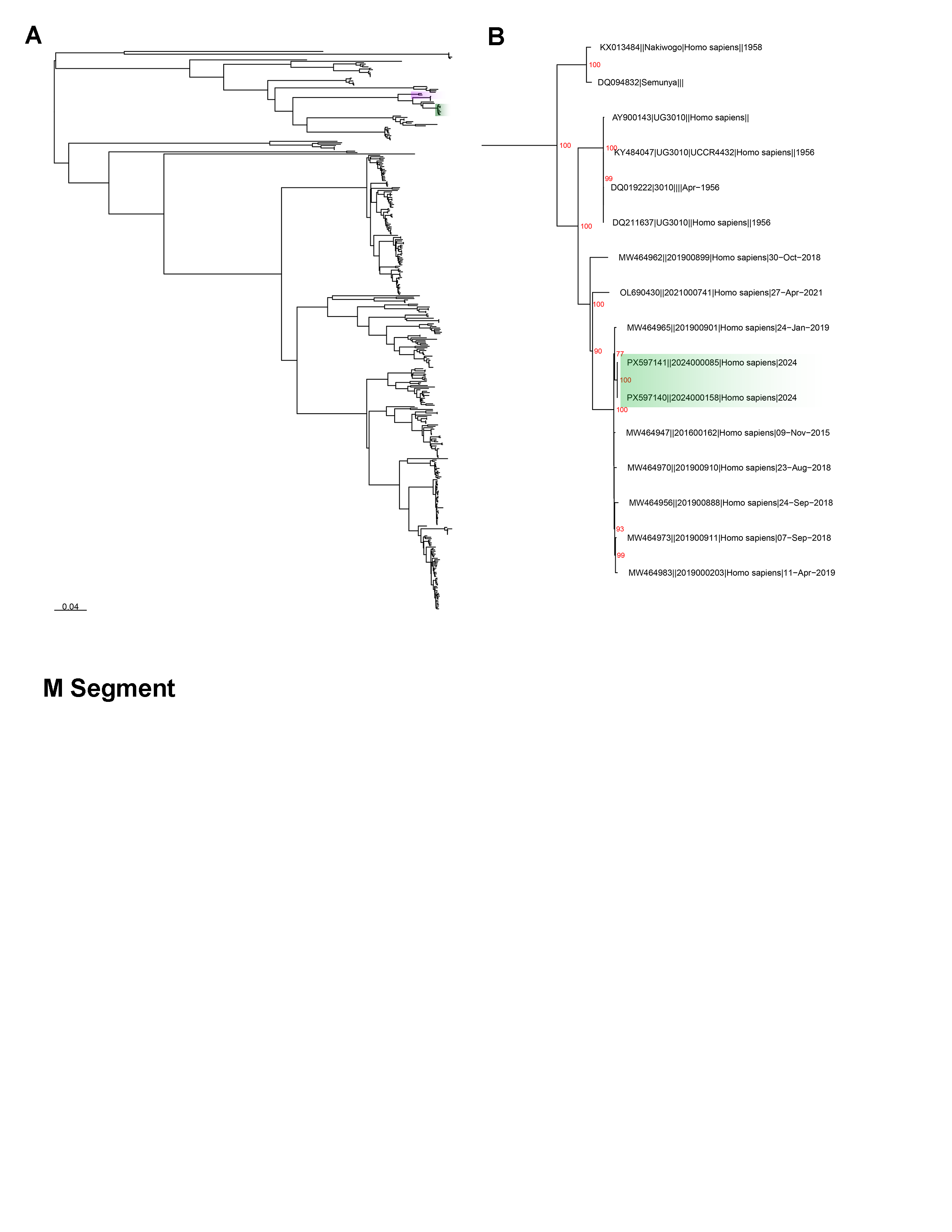


**Supplementary Figure 2.** Maximum-likelihood tree of the L-segment confirming clustering with the 2019 Kiruhura isolate.


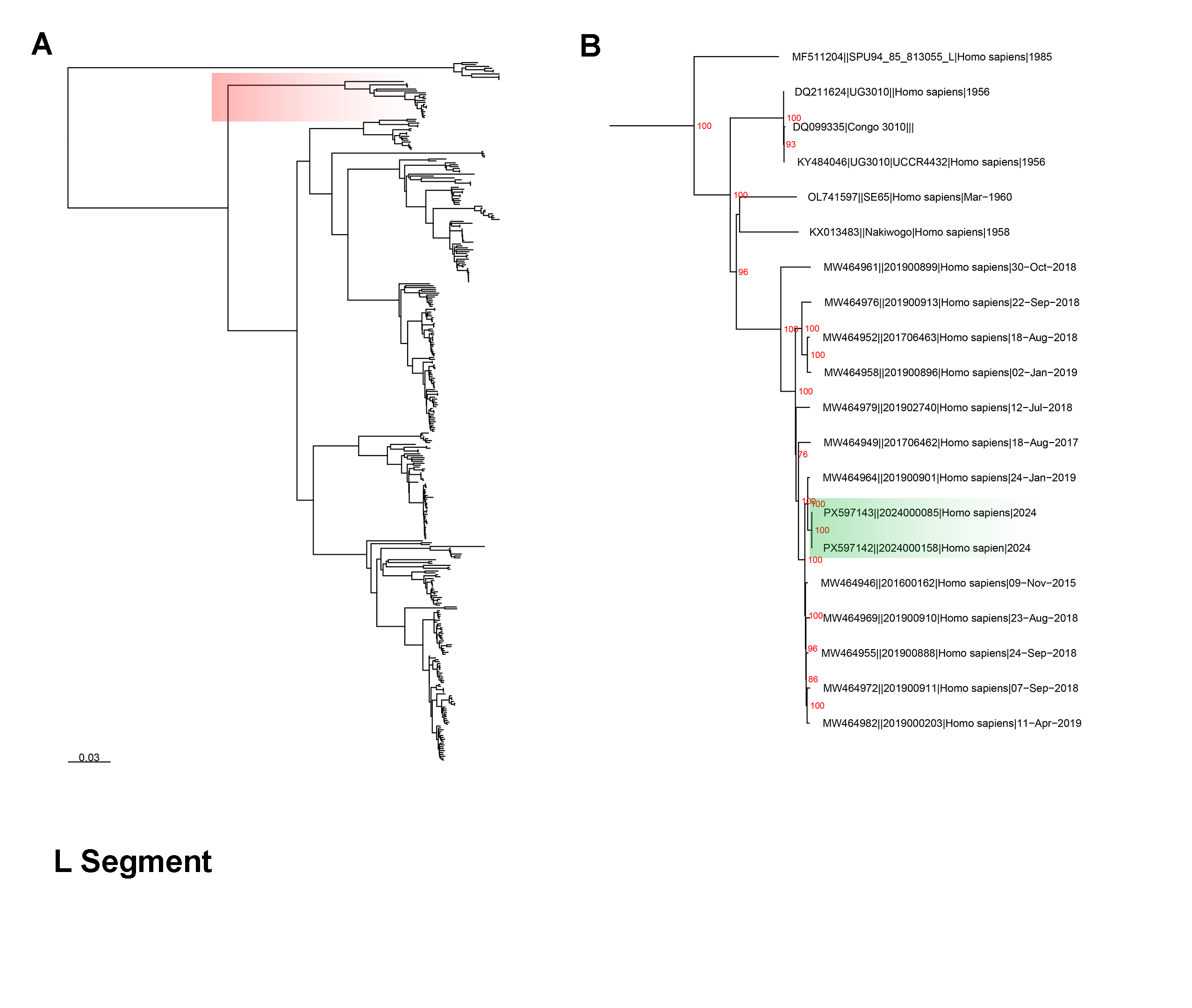

Supplement: Supplementary file 1 — Maximum-likelihood Trees of the M and L segments showing Lyantonde CCHF Virus Sequences within Africa 11. [file mmc1.docx]
